# Microbes in Agriculture: Prospects and Constraints to Their Wider Adoption and Utilization in Nutrient-Poor Environments

**DOI:** 10.3390/microorganisms12112225

**Published:** 2024-11-02

**Authors:** Mustapha Mohammed, Felix D. Dakora

**Affiliations:** 1 Department of Crop Science, University for Development Studies, Tamale P.O. Box TL 1882, Ghana; mmustapha@uds.edu.gh; 2 Department of Chemistry, Tshwane University of Technology, Private Bag X680, Pretoria 0001, South Africa

**Keywords:** plant growth-promoting rhizobacteria, plant-microbe interactions, microbial inoculants, root exudates

## Abstract

Microbes such as bacteria and fungi play important roles in nutrient cycling in soils, often leading to the bioavailability of metabolically important mineral elements such as nitrogen (N), phosphorus (P), iron (Fe), and zinc (Zn). Examples of microbes with beneficial traits for plant growth promotion include mycorrhizal fungi, associative diazotrophs, and the N_2_-fixing rhizobia belonging to the α, β and γ class of Proteobacteria. Mycorrhizal fungi generally contribute to increasing the surface area of soil-root interface for optimum nutrient uptake by plants. However, when transformed into bacteroids inside root nodules, rhizobia also convert N_2_ gas in air into ammonia for use by the bacteria and their host plant. Thus, nodulated legumes can meet a high proportion of their N requirements from N_2_ fixation. The percentage of legume N derived from atmospheric N_2_ fixation varies with crop species and genotype, with reported values ranging from 50–97%, 24–67%, 66–86% 27–92%, 50–92%, and 40–75% for soybean (*Gycine max*), groundnut (*Arachis hypogea*), mung bean (*Vigna radiata*), pigeon pea (*Cajanus cajan*), cowpea (*Vigna unguiculata*), and Kersting’s groundnut (*Macrotyloma geocarpum*), respectively. This suggests that N_2_-fixing legumes require little or no N fertilizer for growth and grain yield when grown under field conditions. Even cereals and other species obtain a substantial proportion of their N nutrition from associative and endophytic N_2_-fixing bacteria. For example, about 12–33% of maize N requirement can be obtained from their association with *Pseudomonas*, *Hebaspirillum*, *Azospirillum,* and *Brevundioronas*, while cucumber can obtain 12.9–20.9% from its interaction with *Paenebacillus beijingensis* BJ-18. Exploiting the plant growth-promoting traits of soil microbes for increased crop productivity without any negative impact on the environment is the basis of green agriculture which is done through the use of biofertilizers. Either alone or in combination with other synergistic rhizobacteria, rhizobia and arbuscular mycorrhizal (AM) fungi have been widely used in agriculture, often increasing crop yields but with occasional failures due to the use of poor-quality inoculants, and wrong application techniques. This review explores the literature regarding the plant growth-promoting traits of soil microbes, and also highlights the bottle-necks in tapping this potential for sustainable agriculture.

## 1. Introduction

With the world’s human population projected to reach over 9 billion by 2050, there is a need for increased agricultural productivity to ensure food and nutritional security [1,2,3]. However, decreasing crop yields due to drought, soil nutrient depletion, pests, and diseases have further threatened global food security [4]. Although the use of chemical fertilizers has been credited for today’s global food and nutritional sufficiency, this has happened at a huge cost to the environment [5]. There is therefore a need to explore greener technologies for greater crop production, especially with a focus on tapping the diverse soil microbes for increased agricultural productivity while minimizing adverse environmental effects [6,7,8]. Microbes such as bacteria and fungi are abundant in soils and possess several traits for improving soil structure and plant growth promotion through nutrient cycling, thus enhancing crop yields [9,10,11]. The mineralization of soil organic matter by soil microbes traditionally increases the bioavailability of nutrient elements such as nitrogen (N), phosphorus (P), potassium (K), and iron (Fe) for uptake by plants [12].

Nitrogen and phosphorus are important nutrient elements known to limit plant growth and therefore require innovative agronomic management to ensure their availability in the rhizosphere for plant uptake in cropping systems [13]. For instance, although N is the most limiting nutrient for plant growth, globally its recovery rate from fertilizers applied to crops is often below 50% due to losses associated with volatilization, leaching, and denitrification [14]. From the early 1890s, when Winogradsky suggested the possible role of the nitrifying bacteria *Nitrosomonas* in agriculture [15], several species of that genus and those of *Nitrobacter* are reported to be nitrifiers [16,17]. While nitrification (the conversion of ammonium to nitrate) generally increases the availability of nitrate for plant uptake, it also produces nitrous oxide (N_2_O), which causes global warming [5]. Additionally, nitrates are easily lost via leaching, thus decreasing nitrogen use efficiency in cropping systems and contributing to groundwater contamination [18]. However, the use of nitrification inhibitors such as 3,4-dimethylpyrazole phosphate can reduce both leaching and nitrous oxide emission in agricultural soils [19]. Furthermore, P use in cropping systems is chemically based and therefore not sustainable. However, the alternative to chemical P fertilizer is rock phosphate, which is declining in reserves [20]. Yet, P is an important component of macromolecules such as adenosine triphosphate (ATP) and ribulose1,5-bisphosphate (RuBP). Therefore, P deficiency in soils can negatively affect plant metabolic processes, including photosynthesis, and thus impair plant growth and grain yield [21].

An alternative to the use of synthetic P fertilizers in agriculture and the problem of declining rock phosphate reserves globally is to tap P-solubilizing soil microbes in cropping systems for enhanced P nutrition by crop species. In contrast to the popular view, there is abundant P in agricultural soils, however, most of it is unavailable to crop plants, as it is bound to Ca, Al, and clay micelles. Various studies (including some from our laboratory) have identified P-solubilizing rhizobia from cowpea, soybean, common bean, Bambara groundnut, etc., that can promote P nutrition in grain legumes [10,22,23,24]. *Bradyrhizobium* sp. TUTNou71 isolated from Bambara groundnut in Mali showed 5-fold more P-solubilizing ability than *Bradyrhizobium* sp. TUTNou73 obtained from the same site, indicating that rhizobia can differ in their P-solubilization efficiency. Similarly, phosphate solubilization varied among soybean rhizobial strains in India, with some isolates showing up to 3-fold higher efficiency [23]. Wekesa et al. [24] also assessed P-solubilization in two common bean rhizobial isolates in Kenya and found marked variation in the trait. Out of 21 cowpea *Bradyrhizobium* isolates from South Africa, only two isolates possessed phosphate-solubilizing ability with near-similar efficiency [22]. These findings suggest the need for identifying high P-solubilizing rhizobia for use as inoculants in cropping systems, especially in degraded soils. However, little is known of P-solubilizing bacteria from cereal crops. Thus, future studies should focus on identifying soil microbes with high P-solubilizing ability for use on cereals.

So far, however, a diverse group of bacteria is known to exhibit plant growth-promoting traits, which include species of the genera *Bacillus*, *Enterobacter,* and *Azospirillum* as well as rhizobia belonging to the α, β, and γ classes of the Proteobacteria [25,26]. The mechanisms of plant growth promotion by soil microbes can range from N_2_ fixation and phosphate solubilization to the synthesis and release of molecules such as siderophores and auxins [26]. Rhizobia are also known to secrete metabolites such as lumichrome, organic acids, vitamins such as riboflavin, and lipo-chito-oligosaccharides (Nod factors) that promote seedling development in legumes [27]. The N-fixed in root nodules are used directly by the bacterial cells for their N nutrition while the surplus is excreted into host plant cells in exchange for photosynthate [28]. The efficiency of the symbiosis can vary with bacterial strain, crop genotype, genotype/strain compatibility, as well as other abiotic factors [29,30]. The symbiotic process can be enhanced by inoculating legumes with elite rhizobial strains or a cocktail of microbes that include non-rhizobial promoters of plant growth [31].

This review discusses the role and mechanisms of plant growth promotion by diverse soil microbes and highlights their potential utilization in nutrient cycling and plant growth promotion, with focus on both N_2_-fixing rhizobia and plant-AM fungi interactions as biofertilizers for increased crop production. The prospects of microbes in the biofortification of crops for improved human nutrition and the challenges to their wider utilization in agriculture are also discussed.

## 2. Overview of Plant–Bacterial Interactions in the Rhizosphere

The term rhizosphere is used to describe the zone of soil that surrounds plant roots. It is usually characterized by a high diversity of bacterial genera and species [32,33] that promote plant growth and adaptation [34]. The synthesis and release of various metabolites by rhizobacteria [27] and their role in the improvement of plant performance has been reviewed by earlier reports [35]. These rhizobacteria comprise species that either enter into intricate symbiotic associations with plants or that exert indirect plant growth promotion via the rhizodeposition of metabolites to enhance the availability of important nutrient elements for plant uptake [36]. Symbiotic rhizobia are, for example, characterized by their ability to colonize root hairs of legumes and induce the formation of nodules which are factories where N_2_ is reduced to NH_3_ by bacteroids and exchanged for plant photosynthate [37]. Rhizobia are therefore the most important soil bacteria in agriculture due to their significant N contribution to cropping systems and natural ecosystems when in association with members of the Leguminosae [38,39].

**Figure 1:**
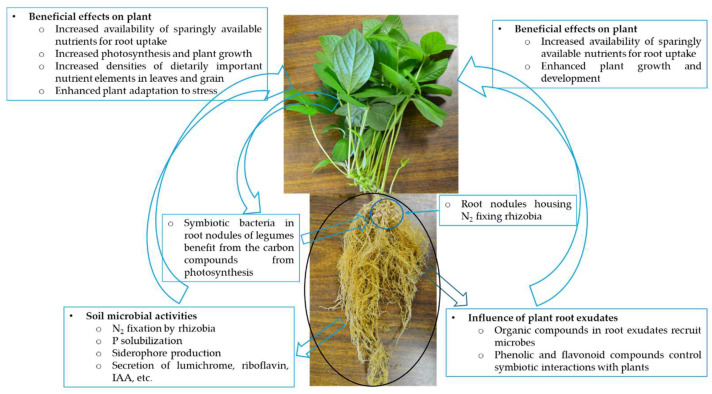
Mechanisms of plant growth promotion by beneficial soil microbes. Rhizodeposition of organic compounds in seed and root exudates is important in shaping soil microbial community structure and activities.

Besides rhizobia, bacterial species belonging to the genera *Bacillus*, *Enterobacter*, *Pseudomonas*, *Azospirillum,* and several others are also abundant in the rhizosphere of plants and contribute to plant growth promotion. However, the mechanisms of plant growth promotion by these rhizobacteria can vary widely, ranging from the production of metabolites such as siderophores, riboflavin, lumichrome, cytokinin, and indole-3-acetic acid to the secretion of various volatile organic compounds, which are all involved in altering plant functioning for improved performance [35] (Figure 1). The production of phytase enzyme by *Bacillus amyloliquefaciens* FZB45, for example, has been reported to promote cabbage growth via improved P nutrition in soils supplemented with phytate [40]. In addition to its plant growth promotion via phosphate solubilization and IAA synthesis, *Bacillus* sp. TZ5 is also capable of bioremediation of cadmium in soils [41]. While rhizobia can exert a direct influence on legume plants through symbiotic N supply, they also promote the growth of non-leguminous plant species via indirect mechanisms [42]. Several non-rhizobial rhizobacteria have been reported to be opportunistic endophytes in root nodules of legumes where they exhibit plant growth-promoting effects [43]. The types and functions of various metabolites employed by bacteria for plant growth promotion have been comprehensively reviewed [35]. Understanding the mechanisms of action of these beneficial rhizobacteria is critical for manipulating them for use as biofertilizers, whether single strain or a cocktail of synergistic bacteria that can improve plant fitness and growth performance [4,12].

## 3. Beneficial Soil Microbes and Plant Growth in Adverse Environments

The rhizosphere of plants generally consists of a cosmopolitan group of microorganisms that exert significant influence on plant fitness and performance [44]. The interaction between plants and microbial communities such as bacteria or fungi can yield beneficial or detrimental outcomes for one or both partners [45,46]. Plants have therefore evolved multiple mechanisms in their interactions with both beneficial and pathogenic microbes within the soil environment. Through the secretion of seed or root exudates which contain a myriad of compounds, plants are able to shape the composition of rhizosphere microbial communities by recruiting those that are beneficial in their interactions while avoiding antagonistic microbes and pathogens [33,47,48,49,50].

Plant root exudates are reported to comprise both low molecular weight compounds (e.g., amino acids, phenolics, and sugars) and high molecular weight macromolecules (e.g., proteins and polysaccharides), which are involved in plant growth promotion and defence [51] (Figure 1). Active rhizodeposition of specific molecules by plant roots generally aims to mobilize microbes capable of alleviating the effects of environmental stresses [52]. The secretion of malic acid by *Arabidopsis thaliana* L. is reported to favour the recruitment of *Bacillus subtilis* in response to foliar infection by pathogenic *Pseudomonas syringae* [53]. Furthermore, *Bacillus amyloliquefaciens* was also found to suppress the growth of phytopathogens, while stimulating plant growth via the synthesis and release of volatile organic compounds such as 2,3-butanedione, 3-hydroxy-2-butanone, 2-propanone, and 2-methylpyridine in a dose-dependent manner [54].

Deficiencies in soil nutrients can also stimulate rhizosphere build-up of specific microbes in order to mitigate such stresses [55]. For example, legumes growing in low-N soils tend to release flavonoid compounds that can chemo-attract beneficial microbes and induce *nod*-genes in symbiotic soil rhizobia, leading to nodule formation and N_2_ fixation in order to alleviate the negative effect of low endogenous soil N on plant growth [37]. Conversely, an increase in N supply to legumes is known to reduce their dependence on symbiotic N for their N nutrition through a decrease in the nitrogenase activity of root nodules [56]. The reduced nitrogenase activity is often attributed to nitrite accumulation from nitrate-reduction, a product that can form nitrosyl-haemoglobin and thus reduce O_2_ diffusion to respiring bacteroids [57,58]. Similarly, in low nutrient soils, mycorrhizal symbiosis can mobilize N, P, Fe, Mn, and Zn for improved plant growth and reproductive performance [59,60]. Microbial interactions with plants can also modify soil structure, leading to increased nutrient availability and uptake, and hence improved plant growth. Plant-microbe interactions are therefore a source of complex mechanisms that have evolved to promote the fitness of both partners under adverse environmental conditions [52].

## 4. Evolution of the Legume-Rhizobia Symbiosis for Promoting Plant Growth

Of the diverse rhizobacteria found in plant rhizospheres, rhizobia are a special group that has evolved the ability to convert atmospheric N_2_ to NH_3_ via the acquisition of nodulation and symbiotic genes from other soil bacteria [61]. From supplying fixed N to their host plants, symbiotic rhizobia have a direct effect on plant growth promotion. The symbiotic mutualism between legumes and rhizobia involves the exchange of molecular signals between the two partners [62,63]. In this process, flavonoids released by legume roots or seeds as exudates play a key role in the signal exchange between legumes and their microsymbionts [64,65]. As a first step, these flavonoid signals act as chemoattractants in a concentration-dependent manner, leading to recruitment of compatible rhizobia to legume root hairs in the rhizosphere [66]. Because these flavonoids are signals, they are required in much lower nanomolar or micromolar concentrations to induce the expression of nodulation (*nod*) genes in compatible symbiotic rhizobia, leading to the synthesis and secretion of lipo-chito-oligosaccharide molecules or Nod factors by the microsymbiont [67,68,69,70]. However, non-flavonoid compounds such as betaines and aldonic acids can also act as *nod-gene* inducers in alfalfa and lupin rhizobia but usually at relatively higher concentrations when compared to flavonoids [51,66].

Host plant perception of the rhizobial nod factors is reported to induce cellular responses, including calcium (Ca^2+^) spiking at the root hair tip followed by root hair curling or deformation, a process that results in rhizobia being engulfed in the curled root tip, leading to the formation of a plant cell wall-derived infection thread that houses the bacteria [68,71]. Bacterial cells induce mitotic division of cortical cells within the infection thread, to form a nodule primordium [72]. Rhizobia released into the plant cell cytoplasm often differentiate into N_2_-fixing bacteroids, enclosed in plant-derived membranes or symbiosomes [66]. The N_2_-fixing nitrogenase in bacteroids is the enzyme responsible for reducing N_2_ to NH_3_. The joint synthesis of leghaemoglobin by the legume and rhizobial partners ensures a low O_2_ (5–30 nM) environment within the symbiosomes, which is a prerequisite for nitrogenase activity [66,73]. N_2_ fixation is reported to commence from 11–15 days after nodule formation after which the plant starts to benefit from the fixed N from root nodules while in return providing the bacteroids with protection, nutrients, and photosynthate for their growth [66,74]. Additionally, the rhizobial bacteria are also reported to mitigate plant adaptation to environmental stresses such as drought, salinity, pH, and heavy metal contamination [75]. Rhizobial production of siderophores [76], solubilization of phosphate, and synthesis of indole acetic acid all promote growth even in non-legumes [77]. With climate change, the ecological significance of rhizobia has increased significantly with greater interest in exploiting the legume/rhizobia symbiosis for sustainable crop production using commercial inoculants [4].

## 5. Microbes in Crop Biofortification

Malnutrition and micronutrient deficiency are high in Africa, highlighting the need for biofortification of food crops with nutrient elements, especially the micronutrients Iron (Fe), Zinc (Zn), Copper (Cu), and Selenium (Se) for human nutrition/health [78,79]. Mineral density in crops is determined by their concentrations in the soil. Where soils are inherently low in nutrients, especially in Africa, fertilizer application is used to increase uptake and accumulation by crop plants, an approach that raises production costs [80,81]. However, beneficial soil microbes are known to promote the bioavailability of dietarily important micronutrients in the rhizosphere of crop plants, thereby naturally promoting sustainable and cost-effective biofortification [79,82]. Soil microbes known for their role in the biofortification of crops include bacterial and fungal species (Table 1). For example, inoculating wheat with *Bacillus* sp. YAM2 significantly increased the levels of Se in kernels relative to uninoculated control [83], just as legume inoculation with rhizobia enhanced Fe and Zn accumulation in shoots [84]. Nodulated legumes therefore appear to benefit markedly from natural biofortification by symbiotic rhizobia, with potential benefits to succeeding cereal crops rotated with legumes; for example, Lengwati et al. [85] found higher concentrations of Fe, Zn, Mn, and Cu in the grains of maize plants that were planted in rotation after different grain legumes [85].

Fungal species are also known for their role in promoting biofortification in plants through the accumulation of nutrient elements in host plants. Inoculating chickpea with arbuscular mycorrhizal (AM) fungi, for example, increased grain concentration of Fe and Zn [86]. Inoculation of wheat with a mixture of the AM fungus *Glomus claroideum* and selenobacteria (e.g., *Stenotrophomonas* sp. B19, *Enterobacter* sp. B16, *Bacillus* sp. R12, and *Pseudomonas* sp. R8) also increased shoot and grain concentrations of Se, suggesting a synergistic interaction of these microbes in crop biofortification [87,88,89]. With climate change and its effect on the declining food and nutritional security globally, there is a need to explore and exploit beneficial plant–microbe interactions for enhanced biofortification of food crops in order to combat protein–calorie malnutrition and micronutrient deficiency using microbial inoculants (Table 1).

**Table 1:** Examples of beneficial microbes and their roles in the biofortification of plant organs with micronutrients.

Microorganism (s)	Treatment Application	Experimental Condition	Crop	Effect	References
* *Glomus mosseae* isolate 112 BEG	Single strain inoculation, supplemented with different levels of nitrogen (N) and phosphorus (P)	Glasshouse	Lettuce	At low P level, Mycorrhizal lettuce plants accumulate greater copper (Cu), iron (Fe), zinc (Zn), and manganese (Mn) at different N levels	[59]
*Azospirillum brasilense* Ab-V6	Single strain inoculation	Field	Maize	Increased grain Zn, Mn, and Cu concentrations	[90]
*Providencia* sp. PW5 + N_60_P_60_K_60_	Applied as a single strain together with N_60_P_60_K_60_	Field	Wheat	Increased grain protein content by 18.6%Increased grain concentration of Fe, Mn, and Cu	[91]
*Acinetobacter* sp. E6.2, * *Glomus claroideum (synonym: Claroideoglomus claroideum);* * *Glomus claroideum*, *Enterobacter* sp. B16	Single strain and dual inoculation	Glasshouse	Wheat	Increased grain selenium (Se) concentration	[87,89]
* *Funneliformis mosseae* and * *Rhizophagus irregularis*	Single strain and dual inoculation	Field	Chickpea	Increased grain Fe and Zn content	[86]
*Bacillus* sp. YAM2	Applied alone, or together with selenate	Naturally lit wire house	Wheat	Increased Fe and Se concentrations in stems and Kernels	[83]
*Bradyrhizobium japonicum* strain WB74	Applied alone or with 5 mM KNO_3_	Glasshouse	Soybean	Genotype-dependent increase in shoot Mn, Zn, and Fe concentrations	[84]
* *Glomus mosseae* (L.) + *Rhizobium leguminosarum* (L.)	Applied as a mixture and supplemented with different N and P rates	Field	Pea	Increased seed Fe, Cu, Zn, and Mn concentrations	[92]
*Sphingomonas* sp. SaMR12, *Enterobacter* sp. SaCS20	Single strain inoculations	Glasshouse, Hydroponic (in growth chamber)	Rice	Increased concentration of Zn in shoot and grain	[93]
*Anabaena* sp.+ * *Trichoderma viride*	Biofilm formulation, supplemented with N, P and potassium (K)	Field	Rice	Increased grain Fe and Zn concentration	[94]

NB: * indicates arbuscular mycorrhizal fungi (AMF).

## 6. Exploitation of Microbial Inoculants in Agriculture

Microbes in agricultural and natural ecosystems are widely known for their role in nutrient cycling, alteration of soil structure, and plant growth promotion via still-unknown mechanisms [12]. The quest to sustainably increase crop yields while reducing agricultural use of chemical fertilizers has stimulated greater interest in tapping beneficial microbes for agriculture. However, their wider adoption and use would require formulation into bioinoculants containing bacteria, fungi, or their combination that can function synergistically to improve plant growth and increase grain yield [4] (Table 2 and Table 3). N_2_-fixing rhizobia either formulated alone or in combination with other beneficial rhizobacteria and endophytes are reported to stimulate plant growth and increase yields under field conditions [10,95,96,97]. In Africa, where most soils are inherently low in mineral nutrients, especially N, inoculating cowpea with *Bradyrhizobium* strains markedly increased grain yield in Ghana and Mozambique [98]. A similar study involving the inoculation of cowpea and soybean in Ghana resulted in increased grain yield and cash income in a location-dependent manner [95]. Field inoculation of common bean (*Phaseolus vulgaris*) with *Rhizobium tropici* strain CIAT 899 and soybean with *Bradyrhizobium japonicum* strain USDA 110 also increased grain yield and marginal dollar returns in Tanzania, with even higher yields when supplemented with low phosphorus application [99]. Inoculating common beans with either *Rhizobium* sp. strain GT-9 or HB-429 also led to increased nodulation, N_2_ fixation, and grain yield relative to the uninoculated control in Ethiopia [100]. However, the most significant success story in the use of rhizobial inoculants has been the case of Brazil, where the recommended use of elite strains is credited for the remarkable increases in soybean grain yield and the reduced agricultural use of chemical N fertilizers [101,102]. Even where soils contained large populations of native rhizobia, soybean inoculation with a mixture of *B. elkanii* SEMIA 587 and *B. japonicum* SEMIA 5080 was found to increase grain yield over N fertilization and uninoculated control [103].

However, tapping the benefits of soil microbes should not be restricted to only legumes and rhizobia. Inoculation of cereals such as maize and wheat with *Azospirillum brasilense* and *A. lipoferum* has been shown to increase grain yield, an indication of the potential for wider benefits of these rhizobacteria in agriculture [90]. Azospirilla are free-living diazotrophs often associated with the roots of grasses and cereal crops and exhibiting plant growth-promoting traits [104]. Efforts at tapping the benefits of diazotrophs have involved their formulation into multi-strain inoculants containing rhizobia and other rhizobacteria such as *Azospirillum*, *Bacillus,* and *Pseudomonas* [105]. An example is the inoculation of pea plants with an ACC deaminase-producing *Pseudomonas putida* and *Rhizobium leguminosarum,* which stimulated plant growth and increased grain yield [106]. Current efforts at tapping soil microbes for increased agricultural yields would require identifying and evaluating multi-strain inoculants in order to maximize their efficiency in a changing climate.

**Table 2:** Examples of experiments reporting the beneficial effects of bacteria-based inoculation on plant performance under different experimental conditions.

Microorganism (s)	Treatment Application	Experiment Condition	Crop	Effect	References
*Azospirillum brasilense*, *A. lipoferum*	Sole inoculation as single strains	Field	Maize, Wheat	Increased grain yield in maize and wheat	[90]
*Bacillus amyloliquefaciens* FZB45	Applied alone at two rates, or together with four levels of phosphorus (P) in factorial experiment	Growth chamber	Cabbage	Increased plant growth at higher rates of phytate supply	[40]
*Bradyrhizobium* sp. (strain CB 1809 + strain CPAC 7), (strain 29 W + SEMIA 587)	Applied as microbial consortium	Field	Soybean	Increased nodule occupancy Increased grain yield Increased grain N content	[107]
*Bradyrhizobium* sp.	Applied alone, or together with phosphorus (P) or nitrogen (N)	Field	Common bean, Soybean	Inoculation alone increased grain yield Inoculation + P increased grain yield over inoculation alone, and N or P alone	[99]
*Bradyrhizobium* sp. BR 3262 and *Bradyrhizobium* sp. BR 3267	Sole inoculation as single strains	Field	Cowpea	Increased nodulation and plant growth Increased grain yield	[95]
*Bradyrhizobium* strain USDA 110	Sole inoculation, supplemented with P and organic manure	Field	Soybean	Increased nodulation Increased plant growth Increased rainwater use efficiency Increased agronomic P use efficiency Increased grain yield	[108]
*Bradyrhizobium* sp. (76 native African isolates)	Sole inoculation as single strains	Glasshouse	Bambara groundnut	Increased leaf chlorophyll concentration over nitrate-feeding Increased stomatal conductance Increased photosynthetic rates Increased plant growth	[10]
*Bradyrhizobium* sp. (40 native African isolates)	Sole inoculation as single strains	Glasshouse	Kersting’s groundnut	Increased leaf chlorophyll concentration over nitrate-feeding Increased stomatal conductance Increased photosynthetic rates Increased plant growth	[97]
*Bradyrhizobium* sp. (17 native African isolates)	Sole inoculation as single strains	Glasshouse	Cowpea	Increased leaf chlorophyll concentration over nitrate-feeding Increased stomatal conductance Increased photosynthetic rates Increased plant growth	[109]
*Pseudomonas putida* strain PSE3 + *Rhizobium leguminosarum* strain RP2	Applied as microbial consortium	Glasshouse Field	pea	Increased nodulation and leghaemoglobin content of nodules Stimulates plant growth Increased leaf chlorophyll content	[106]
*Rhizobium leguminosarum* strain RP2	Applied alone or together with diammonium phosphate	Glasshouse Field	pea	Increased nodulation and leghaemoglobin content of nodules Stimulates plant growth	[106]
*Rhizobium* sp. strains HB-429	Applied alone, or together with different P levels	Field	Common bean	Increased plant growth Increased N-fixed Increased grain yield	[100]

**Table 3:** Examples of experiments reporting the beneficial effects of fungi-based/fungi + bacteria-based inoculation on plant performance under different experimental conditions.

Microorganism (s)	Treatment Application	Experiment Condition	Crop	Effect	References
*Aspergillus* sp. NPF7	Sole inoculation	Growth chamber	Chickpea, Wheat	Stimulated germination Increased plant growth via the synthesis of phytohormones (e.g., Indole-3-acetic acid (IAA), siderophore, gibberellic, phosphate solubilization)	[110]
* *Funneliformis mosseae*, * *Rhizophagus irregularis*	Single-strain inoculation or dual inoculation	Field	Chickpea	Increased plant growth Increased grain yield	[86]
* Glomus intraradices BEG 123 and * G. viscosum 126	Sole inoculation as single strains	Glasshouse	Olive	Increased plant growth of two olive cultivars	[111]
* G. deserticola, * G. spp. (*G. claroideum*, *G. etunicatum*, *G. geosporum*, *G. intraradices*, *G. mosseae*)	Applied as a microbial mixture. Field soil was also used as a control	Glasshouse	Maize	Increased plant growth compared to control (field soil)	[112]
* *Glomus* sp. LPA21, Commercial * *Glomus* sp. (AGC or Phytotec)	Inoculation at different rates of 1–5% (*w*/*w*)	Glasshouse	Grapevine, Pineapple	Increased shoot and root growth relative to control	[113]
* *Glomus intraradices*, *Rhizobium tropici* CIAT899	Dual inoculation	Glasshouse	Common bean	Increased nodulation Promotes shoot and root growth compared to control or single inoculants Increased shoot N and P accumulation compared to control or single inoculants	[114]
* *Glomus fasciculatum* + *Azotobacter chroococcum* + *Bacillus* sp.	Applied as a microbial consortium	Field	Wheat	Increased plant growth Increased grain yield	[115]
* *Glomus intraradices*	Single-strain inoculation at different levels of salinity and P	Field	Pepper	Mycorrhizal inoculation increased plant growth at all salinity levels	[116]
* *G. mosseae*, *Bradyrhizobium* sp. BXYD3	Single strain inoculation or co-inoculation; supplanted with N, P, and potassium (K)	Field, Glasshouse	Soybean	Increased plant growth Increased N and P content of plants	[117]
*Phoma* sp. GAH7	Sole inoculation	Glasshouse	Cucumber	Increased plant height Increased plant weight	[118]
* *Rhizophagus irregularis* DAOM 197198	Sole inoculation, Uninoculated plots as control	Field	Potato	Increased tuber yield	[119]
* *Trichoderma virens*, * *Trichoderma atroviride*	Single strain inoculation	Axenic conditions	Arabidopsis	Stimulates lateral root growth Increased biomass accumulation	[120]

NB: * indicate arbuscular mycorrhizal fungi (AMF).

## 7. Constraints to the Wider Exploitation of Microbial Inoculants

The past decades have seen significant research and commercial interest in the use of microbial inoculants as eco-friendly technologies for sustainable crop productivity [95,100,101,102,121]. As a result, large amounts of data have been generated to aid our understanding of how beneficial soil microbes such as bacteria and fungi occupy centre stage in the maintenance of plant fitness and productivity. The potential positive impact of microbial inoculants as components of sustainable crop production systems was recently reviewed by Shahwar et al. [122]. Despite some of the successes in increasing crop yields using biostimulants from soil microbes, their wider adoption is constrained by several factors [123].

### 7.1. Biotic and Abiotic Factors

Firstly, the inherent susceptibility of biological processes to biotic and abiotic stresses often causes inconsistent outcomes from the use of the same inoculant over time and, space, which is a major obstacle to the adoption of these green technologies [124]. With rhizobial inoculants for example, the presence of large populations of ineffective but highly competitive native strains can cause the failure of highly effective inoculant strains to nodulate the host plant [123]. This challenge is compounded by the fact that, even in the same environment, inoculation response can vary with legume genotype [96]. For example, despite the known plant growth-promoting effect of *Azospirillum* sp., its co-inoculation failed to increase soybean yields in Mozambican soils, prompting the need for further research to harness the benefits of these plant–bacterial interactions in changing environments [11]. Moreover, tapping the multiple beneficial traits of diverse microbes through their formulation into a multi-strain inoculant sometimes fails due to possible incompatibility among the different components [125]. For instance, the co-inoculation of *Phoma* sp. GS8-2 or GS8-3 with the AM fungus *Glomus mosseae* decreased the level of disease resistance conferred by single strain inoculation with either *Phoma* isolate [126]. Thus, the formulation of many microbes into a single inoculant often requires an understanding of their mechanisms of action in order to select those that present synergistic interactions to aid overall plant growth and productivity. The fact that the persistence of AM fungal inoculants in soils can be location-specific is a major setback in predicting the performance of such inoculants in the field [127], suggesting a need to explore soils for effective native strains that can be harnessed for increased plant performance [111].

### 7.2. Quality Control Issues

Nevertheless, the quality of the formulation can also be a factor hindering inoculant performance in the field [128]. In the absence of quality control, the proliferation of poor-quality inoculants containing fewer than optimum bacterial cells can lead to inoculation failure, and thus deter farmers’ adoption due to poor performance [123]. Quality controls are therefore often instituted and standards can vary among countries [128], as found in Spain and France, which have regulations for safeguarding the quality of biofertilizers used by farmers [129]. Canada and Australia are also producing rhizobial inoculants that contain recommended numbers of viable cells and are free of contaminants [123,130]. Furthermore, the identification of inoculant strains that are suited for multiple environments is also a challenge, prompting more research aimed at producing multi-strain inoculants for use in different environments [31].

### 7.3. Limited Shelf Life

The shelf life of inoculants is equally important and critical for achieving inoculation success, and it is thus a constraint to the adoption and utilization of microbial inoculants [131], especially among rural farmers who may lack the appropriate storage facilities. Factors affecting inoculant shelf life include the type of carrier used, temperature, moisture, storage time, microbial strain, and their interactions [132,133]. For example, the use of charcoal-soil mixture as a carrier by Gaind and Gaur [132] retained a greater number of viable phosphate-solubilizing *Pseudomonas* cells than the use of paddy straw compost. Biradar and Santhosh [134] also found that the cell population and viability of *Pseudomonas fluorescens* were greater when polyvinlypyrrolidone (PVP, 2%) was used as cell protectant along with the use of adjuvants, surfactant, and preservative, resulting in 1.76 × 10^10^ CFU/mL at 28 °C after 180 days. Amending a liquid *Rhizobium* sp. strain MB1503 with 1% or 2% PVP produced a higher viable cell count which led to enhanced plant growth and nitrogen content of mung bean (*Vigna radiata* L.) [135]. Given the cell survival and shelf life constraints of inoculants, it is recommended to provide information about the optimum storage and handling conditions on inoculant sachets [136].

## 8. Future Perspectives

For millennia, soil microbes have been known for their beneficial contribution to agriculture and natural ecosystems, which has led to significant research into their diversity, distribution, and mechanisms of action. Key areas for future research should include (i) bioprospecting for rhizobial strains with high N_2_-fixing ability, (ii) identifying rhizobia with multiple beneficial traits such as P-solubilization, IAA secretion, siderophores production, drought, and salinity tolerance, as well as low pH resistance, to enable their use as inoculants in multiple and diverse environments, (iii) exploring soil microbes with the ability to enhance the accumulation of dietarily important trace elements in both legume and cereal crops, (iv) promoting the development of multi-strain and multi-species microbial inoculants for use in harsh and difficult environments.

While research on the legume/rhizobia symbiosis has produced technologies that have promoted crop productivity and restored vegetation to arid and degraded environments, less has been done on tapping the associative symbiosis commonly found in cereal/microbe interactions. Many tropical pasture grasses such as *Digitaria decumbence* Stent, often produce dark-green foliage reminiscent of nodulated legumes. Many associative N_2_-fixing bacteria such as *Herbaspirillum seropedicaea* strain (ATCC) 35892, *Pseudomonas jessenii* strain CIP105274 [137], *Enterobacter doacae* [138], *Pseudomonas*, *Bacillus*, *Burkholderia*, *Pantoea* [139], and *Klebsiella variicola* [140] have been found in sorghum, water yam, wheat, sugarcane, sweet potato, etc. Apparently, N_2_-fixing bacteria such as *Pseudomonas*, *Hebaspirillum*, *Azospirillum,* and *Brevundioronas*, can provide 12–33% of total N to maize [141], while *Paenebacillus beijingensis* BJ-18 provided 12.9–20.9% to cucumber through biological N_2_ fixation [142].

With climate change and the effect of synthetic N in agriculture on global warming from agricultural use, there is a renewed effort to exploit the cereal/microbe interaction. In addition to providing biologically fixed N to plants, some of these associative diazotrophs especially endophytes also promote plant growth via the synthesis and release of plant hormones such as indole acetic acid, cytokinins, and gibberellins, which promote plant growth via enhanced root branching and elongation, increased root hairs density and greater absorption of water and nutrients [104,143]. The recent discovery that inoculating cassava with a *Curtobacterium* endophyte respectively increased root, stem, and leaf biomass by 17.6%, 12.6%, and 10.3% further stresses the need for intensified research into non-rhizobial plant–microbe interactions; the observed increases in the biomass of cassava plants were attributed to biological N_2_ fixation, secretion of indole-3-acetic acid and P-solubilization [144]. Crop plants such as sugarcane, cassava, yam, taro, etc. that are a huge reservoir of sugar and carbohydrates should be targeted in bioprospecting for N_2_-fixing and plant growth-promoting endophytes, as they are an easy source of energy for N_2_ fixation and the biosynthesis of growth stimulating metabolites.

## 9. Concluding Remarks

The abundance of beneficial microbes in soils offers a great opportunity for developing greener technologies to replace chemical-based crop production systems. The multiple roles played by soil microbes in cropping systems and nature conservation require continued research. The role of microbes in the biofortification of food crops should be pursued vigorously to avoid food insecurity and hidden hunger, especially among poorer populations across the world. Tapping beneficial microbes for a transformed global agricultural system while eliminating chemically based approaches has a high of reducing agriculture’s contribution to climate change.

## Data Availability

All data/information used in this review are presented in the paper and the sources are accordingly cited and referenced.
